# Barriers and Facilitators for Accessing Oral Health Care for Ukrainian Newcomers to Nova Scotia

**DOI:** 10.1177/23800844251352395

**Published:** 2025-07-17

**Authors:** H. Doucette, Y. Tylchak, S. Saad, V. D’Souza

**Affiliations:** 1 Faculty of Dentistry, Dalhousie University, Halifax, Nova Scotia, Canada; 2 American Chamber of Commerce in Ukraine, Kyiv, Ukarain, Ukraine; 3 Faculty of Dentistry, University of Toronto, Toronto, Ontario, Canada; 4 Department of Dental Clinical Sciences, Faculty of Dentistry, Dalhousie University, Halifax, Nova Scotia, Canada; 5 Department of Surgery, Faculty of Medicine, Dalhousie University, Halifax, Nova Scotia, Canada; 6 Nova Scotia Health Authority, Beatrice Hunter Cancer Research Institute, Healthy Populations Institute, Halifax, Nova Scotia, Canada

**Keywords:** Refugees, Health knowledge, Language, Acculturation, Health insurance, Canada

## Abstract

**Introduction:**

The war in Ukraine has resulted in a mass exodus of Ukrainians fleeing their country and seeking resettlement in many countries, including Canada. There is a lack of literature, particularly qualitative, that explores past experiences with oral health care in the country of origin for newcomers and the experience of access and utilization of oral health care once in Canada. The increase in Ukrainian newcomers to Canada requires an exploration of barriers and facilitators to oral health care access to inform policy.

**Objective:**

To explore the barriers and facilitators to oral health care experienced by Ukrainian newcomers during resettlement in Nova Scotia, Canada.

**Methods:**

This study used a narrative qualitative methodology. Adult Ukrainian newcomers who arrived in Canada after the Russian invasion in 2022 were recruited via social media and through recruitment flyers advertised at the Immigrant Services Association of Nova Scotia. They were interviewed between February 17 and July 1, 2023, in the Ukrainian language. The interviews were audio recorded, transcribed, and analyzed via inductive and deductive line-by-line coding per the thematic analysis method. Codes were grouped to form categories and themes.

**Results:**

Participants identified facilitators to oral health care that included friends and family, social networks, and information provided through the workplace. Barriers to access included cost, referral process, location and wait times for specialists, language, and lack of understanding the oral health care and dental insurance systems in Canada.

**Conclusion:**

Ukrainian newcomers to Nova Scotia face several barriers to oral health care access. Interventions to address these barriers should be considered to ensure equitable access to oral health care services during the resettlement process.

**Knowledge Transfer Statement:**

The results of this study may be used to inform policy to facilitate timely access to oral health care for Ukrainian newcomers. The findings may also guide professionals when providing oral health care services to Ukrainian newcomers. Addressing the barriers and maximizing the facilitators to oral health care services for this population could help to improve access to oral health care services and oral health outcomes.

## Introduction

Poor oral health can interfere with one’s ability to eat, sleep, speak, and socialize with others, leading to social isolation and negatively affecting quality of life. Additionally, it can exacerbate systemic health conditions and increase hospitalizations and health care expenditures. As such, it is important to highlight the importance of regular preventative oral health care and timely access to emergency and restorative oral health care when oral health issues arise (Watt 2005).

The well-being of a population is complex and affected mainly by health with a variety of factors, including but not limited to the lived experience of past and present social and structural determinants of health (Petersen and Kwan 2011). Vulnerable populations across Canada, including new immigrants, have been identified as facing challenges accessing regular oral health care. New immigrants to Canada experience a significant increase in self-reported oral health issues during the first 4 y of resettlement, indicating a need for an exploration of factors affecting access to oral health care and the development of policies and programs focused on improving access to oral health care for this population (Calvasina et al. 2015). While there is limited research on the oral health status of new immigrants in Nova Scotia, Ghiabi et al. (2014) reported a high level of untreated caries and periodontal disease among new immigrants and Bhutanese refugees in Nova Scotia, despite many participants self-reporting positive perceptions of their oral health.

Russia’s war against Ukraine has resulted in a mass exodus of Ukrainians fleeing their country and seeking resettlement in many countries, including Canada. As of February 2024, Canada had aided >958,190 Ukrainian nationals and their families by approving temporary residence applications as part of the Canada-Ukraine Authorization for Emergency Travel (Xhardez and Soennecken 2023). This program provides expedited work and study visa processing, financial aid, provincial health coverage, and access to federal support programs, demonstrating Canada’s commitment to assisting those affected by the Russian invasion of Ukraine (Xhardez and Soennecken 2023).

In Nova Scotia, 2 organizations—Immigrant Services Association of Nova Scotia (ISANS) and the Young Men’s Christian Association (YMCA)—are working closely with government and community partners to welcome and provide support to Ukrainians arriving in the province (Nova Scotia Immigration 2024). Ukrainians and their families arriving in Nova Scotia with open work permits are eligible to apply for Nova Scotia Medical Service Insurance to enable access to publicly funded health care (Nova Scotia Immigration 2024). While Medical Service Insurance provides basic dental care coverage for children aged ≤15 y, currently there is no financial coverage for routine dental services for adult newcomers (Fierlbeck 2018). Although Canada has recently implemented the Canadian Dental Care Plan, newcomers do not qualify for this program as it requires individuals to have filed an income tax return in Canada the previous year (Government of Canada 2024).

Despite the recent increase in Ukrainian newcomers in Nova Scotia, there is a lack of literature on the experience of navigating access to oral health care during the resettlement process from the perspective of this population. As access to oral health care is related to oral health outcomes and therefore quality of life, it is important to understand a population’s experience with accessing oral health care. As such, an exploration of barriers and facilitators of access to oral health care for Ukrainian newcomers in Nova Scotia is required to inform oral health policy and practice to ensure access to preventative oral health care and treatment for oral health disease. Moreover, understanding the barriers and facilitators of access to oral health care in the country of origin is essential for comprehensively assessing the oral care experiences of ukrainian newcomers; furthermore, examining the potential impact of such barriers and facilitators may permit us to anticipate how these experiences may influence approaches to accessing oral health care in Canada.

## Methods

### Methodology and Study Setting

Ethics approval was obtained from the Research Ethics Board of Dalhousie University (REB file 2022-6286). This study was conducted by utilizing descriptive qualitative research methodology (Sandelowski 2000) at the Faculty of Dentistry, Dalhousie University, Halifax, Nova Scotia. Descriptive qualitative methodology provides an opportunity to capture in-depth lived experiences of individuals. The epistemological framework of this study design lies within the naturalistic inquiry. While it is considered one of the least theoretical qualitative inquiries, it allows researchers to explore specific research questions and interpret the observations of specific people in a social and cultural context (Neergaard et al. 2009).

### Positionality

It is essential to understand the positionality of the researchers, as well as their background and work experience on the topic. The first author (H.D.), with a formal background in dental hygiene, is the director of the Newcomer Oral Health Clinic and has conducted research involving new immigrants and refugees. The last author (V.D.) is a dental public health specialist who is involved in research on marginalized populations and their access to dental care services. Y.T. is a masters student in healthcare management and a Ukrainian newcomer in Nova Scotia who understands the cultural and systemic challenges in accessing dental services in Canada and Ukraine and provided insight into developing the interview guide, identifying and recruiting study participants, and conducting interviews and data analyses. The fourth researcher, S.S., is a dental student and an immigrant to Canada who understands the challenges faced by newcomers when accessing oral health care.

### Trustworthiness and Rigor

Trustworthiness was established through 1) credibility by member checking transcripts for accuracy and 2) confirmability by maintaining a detailed audit trail of the coding process while confirming and validating by the research team members.

### Participants

The study participants were Ukrainian newcomers to Nova Scotia who fled Ukraine due to the Russian invasion in 2022, had been living in Canada for at least 12 wk, and were aged ≥18 y. Participants were able to self-recruit through the recruitment flyer that was posted in social media groups whose target audience was Ukrainian newcomers. Also, the ISANS Ukrainian Project team lead provided a study information flyer to potential participants at the ISANS office, and if they expressed interested, their names and contact information were provided to the designated research team member (Y.T.).

### Recruitment and Data Collection

Participants were screened and selected via a purposive sampling method to include those from different age groups, genders, and family family status. All recruited participants were invited for in-depth semistructured interviews at the Faculty of Dentistry, were required to provide written informed consent prior to their participation in the study, and were given pseudonyms to maintain their anonymity. Recruitment continued until the content of the collected data reached saturation.

Data were collected through semistructured in-depth interviews via an interview guide (Table 1) created by the research team, which features oral health professionals with experience in conducting qualitative research. All interviews were conducted in person, in Ukrainian, by Y.T. in a private room at the Faculty of Dentistry, Dalhousie University, and were audio recorded with a Sony ICDPX370 IC voice recorder.

**Table 1:** General Characteristics of the Population Sample.

Pseudonyms	Age	Sex	Employment status	Education level	Marital status	Arrival date in Canada	Dental visit pattern	No. of dental visits since arrived	Reason for dental visit	Has a dental insurance?	Avoided dental care due to cost
Kateryna	61	F	Not employed	Masters degree	Divorced	2022 April	Annually	1	Examination, restoration, debridement	No	No
Solomiia	32	F	Not employed	Masters degree	Married	2022 May	Bi-annually	1	Examination, restoration	No	No
Ostap	21	M	Not employed	High school diploma	Married	2022 Aug	Bi-annually	1	Examination, restoration, wisdom teeth extractions	No	No
Ihor	36	M	Employed	Masters degree	Divorced	2022 Nov	Annually	No	NA	Yes	No
Oksana	38	F	Not employed	Masters degree	Married	2022 May	Bi-annually	No	Scheduled	Yes	Yes
Andriy	66	M	Employed	Masters degree	Married	2022 Sept	Only if had a problem	No visits	No	No	No
Yuliia	57	F	Employed	Masters degree	Widowed	2022 April	Annually	2 visits	Examination, debridement	Yes	No
Maksym	44	M	Employed	Bachelors degree	Married	2022 June	If had pain	A few visits	Examination, restorations, root canal treatment	Yes	No
Sofiia	42	F	Employed	Masters degree	Married	2022 June	If had pain	A few visits	Examination, restorations, extractions	Yes	Yes
Ivan	25	M	Employed	Bachelors degree	Married	2022 Dec	Annually	A few visits	Examination, restorations	Yes	Yes

Interview questions were aimed to better understand the past and present lived experiences of Ukrainian newcomers to Nova Scotia and their knowledge about oral health, their oral health care experience, and their utilization of oral health care services. Y.T., a bilingual research team member fluent in Ukrainian and English, conducted the interviews with conscious effort to provide a nonjudgmental environment that was conducive to the free flow of thoughts and description of experiences, while remaining respectful of the sensitive nature of the conversations. In addition, supplementary memos were written after each interview to chronicle the researcher’s reflections and perceptions of the interview. Also, participants’ limited sociodemographic information was collected.

### Data Analysis

The audiotape recordings were transcribed verbatim in Ukrainian and then translated into English by Y.T. The transcripts were read and reread by 3 research team members (H.D., Y.T., S.S.) and then coded independently by 2 research team members (Y.T. and S.S.) using the line-by-line inductive and deductive coding method. For the inductive coding, the 2 reviewers coded a sample text and verified the coding with each other to achieve consistency. The reviewers then used the same process to code the entire text, repeatedly assessing the coding’s consistency by going back and forth and confirming the process (O’Brien et al. 2014). A third research team member (H.D.) confirmed the consistency of the codes. Codes were merged to produce categories and subcategories and were confirmed by the research team. The Standards for Reporting Qualitative Research checklist was used to ensure the quality of reporting the study (O’Brien et al. 2014).

## Results

Of the 31 potential participants who expressed interest in participating in the study, 10 were purposefully selected: 5 females and 5 males with ages ranging from 21 to 66 y. Sociodemographic information was collected (Table 2). Nine had university degrees, 6 were employed (5 of whom had dental insurance benefits), and 7 had accessed oral health care services since their arrival in Nova Scotia. Interviews were approximately 40 min, ranging from 20 to 60 min.

**Table 2.** General Characteristics of the Sample.

Proxy Name	Age, y	Sex	Employment Status	Education Level	Marital Status	Arrived in Canada	Dental Visit Pattern	Dental Visits in Canada	Reason for Dental Visit in Canada	Dental Insurance Status	Avoided Dental Care due to Cost
Kateryna	61	F	Not employed	Master	Divorced	Apr 2022	Annually	1 visit	Examination, filling, cleaning	No	No
Solomiia	32	F	Not employed	Master	Married	May 2022	Biannually	1 visit	Examination and filling	No	No
Ostap	21	M	Not employed	High school	Married	Aug 2022	Biannually	1 visit	Examination, filling, and wisdom teeth removal	No	No
Ihor	36	M	Employed	Master	Divorced	Nov 2022	Annually	None	—	Yes	No
Oksana	38	F	Not employed	Master	Married	May 2022	Biannually	None	Scheduled	Yes	Yes
Andriy	66	M	Employed	Master	Married	Sept 2022	Only if had a problem	None	—	No	No
Yuliia	57	F	Employed	Master	Widowed	Apr 2022	Annually	2 visits	Examination and cleaning	Yes	No
Maksym	44	M	Employed	Bachelor	Married	Jun 2022	If had pain	A few visits	Examination, filling, and root canal	Yes	No
Sofiia	42	F	Employed	Master	Married	Jun 2022	If had pain	A few visits	Examination, fillings, and extraction	Yes	Yes
Ivan	25	M	Employed	Bachelor	Married	Dec 2022	Annually	A few visits	Examination and fillings	Yes	Yes

F, female; M, male.

Five categories were identified in the data analysis (Table 3):

1) Barriers for accessing oral health care services in Ukraine: war, past traumatic oral health care experiences, cost, and limited facilities
2) Barriers for accessing oral health care services in Canada: cost, scheduling and wait times, lack of understanding the oral care health care system in Canada, lack of knowledge of the dental insurance system, lack of information for oral health care resources, and language and cultural barriers
3) Facilitators for accessing oral health care services in Ukraine: ease of scheduling appointments, affordability, oral health care culture shift after Soviet Union times, full-service dental offices
4) Facilitators for accessing oral health care services in Canada: availability of dental insurance, social networks, location of dental clinics, availability of interpreters
5) Suggestions for improvements in access to oral health care services for newcomers in Nova Scotia: financial assistance, informing Ukrainians about the oral health care system in Canada, receiving oral health care with assistance of intermediates, recruitment of Ukrainian oral health care providers (OHCPs)

**Table 3.** Categories and Subcategories.

1. Barriers to oral health care access in Ukraine 1.1. War 1.2. Past traumatic oral health care experiences 1.3. Cost 1.4. Limited facilities
2. Barriers to oral health care access in Canada 2.1. Cost 2.2. Scheduling and wait times 2.3. Lack of understanding of oral health care system 2.4. Lack of knowledge of dental insurance system 2.5. Lack of information for oral health care resources 2.6. Language-culture
3. Facilitators for accessing oral Health care services in Ukraine 3.1. Ease of scheduling oral health care appointments 3.2. Affordability 3.3. Oral health care culture shift after Soviet Union times 3.4. Full-service dental offices
4. Facilitators for access to oral health care services in Canada 4.1. Dental insurance benefits 4.2. Social networks, location of dental offices, and interpreters
5. Suggestions for improving oral health care access for newcomers 5.1. Discounts 5.2. Cost-free or reduced-fee consultation 5.3. Informing Ukrainians about oral health care system in Canada 5.4. Receiving oral health care with assistance of intermediates 5.5. Recruitment of Ukrainian oral health care providers

These categories are described in turn, with corresponding quotes of the participants.

### Barriers to Oral Health Care Access in Ukraine

To provide context to the participants’ experience with oral health care services in Nova Scotia, their past experiences with accessing oral health care services in Ukraine were explored. The most frequently observed barriers to oral health care services included war-related issues, past traumatic oral health care experiences, costs, and limited access to specific services.

#### War

The war between Russia and Ukraine added significant challenges to oral health care utilization for many, resulting in disruption of and inability to complete dental procedures already in progress, such as dental implants, prosthetics, and crowns. Oksana required a crown for 1 tooth. Although her tooth was prepared and the crown was fabricated, she was unable to have the crown placed prior to fleeing Ukraine. She explained,

I needed to replace a dental crown. Because of the war I am today in Canada with this crown. It has not been placed yet [the crown was fabricated and given to her]. Maybe it does not make sense to use it. In addition, the cavity still exists in one of my posterior teeth for this crown. I did not have time to complete the process.

Her preventive oral health care visits were also affected. She continued, “We scheduled a planned visit, which should be once every six months. But during last year and due to evacuation, we missed our previous ones [appointments].”

Logistical difficulties in traveling to different cities for referred dental appointments, concerns surrounding personal safety, power outages, and energy infrastructure damages were significant. Kateryna described her feelings of fear:

[Before the war] I received the referral to periodontist. But I needed to go to Kramatorks, another town. I didn’t have the possibility because of the war. I planned to go after New Year. And in February the war started. I could not stay more time in Ukraine, it was scary. The martial law was announced. One day, I was outside and saw the rocket above my head. I even sat down.

Some could not maintain their appointments due to the power outages. Ihor explained, “We had problems with electricity. It was needed to catch the power [have a visit when there was electricity] to get an appointment. When I visited the doctor, his clinic [office] didn’t have the generator.”

#### Past Traumatic Oral Health Experiences

Participants aged ≥38 y described their oral health care experiences during Soviet Union times as painful and traumatic. This included completing dental procedures without anesthesia, lack of empathy from dentists, and limited or poor quality equipment due to the state system of ownership. These past negative experiences resulted in fear and avoidance of regular dental visits and extensive oral health care needs. Oksana reported,

In Soviet Union pediatric clinics dentists did not communicate with children properly during the visits. These doctors did not explain to the child psychologically which dental treatment was planned to be done. I experienced a lot of pain during the visits. Sometimes, I could not tolerate pain during the treatment. I asked them to stop the dental machine for some moments. But they did not listen to me. As a result, I did not proceed with treatment for my other dental problems, lost my teeth and needed to have root canal treatment.

#### Cost

Although most participants found oral health care affordable in Ukraine, 2 felt that it was relatively expensive (i.e., based on the average income). Yuliia stated,

In Ukraine, it is believed that dental treatment is expensive. During the last few years, the prices have risen. With my income, it was expensive. My salary was US $400 [per month]. It was enough to cover living expenses, but I lacked money for two things—a car and dental treatment. It was difficult to cover the dental crown, cleaning, and filling expenses together.

#### Limited Facilities

In Ukraine, limited access to specific dental equipment was a barrier to oral health care utilization. Some dental offices did not have radiographic equipment, and as a result, patients had to go to dental offices that had such equipment, resulting in inconvenience and added expense. Sofiia stated, “Only one clinic [office] in my town had x-ray [equipment]. If you visit another one, you need to cross all the town, do the x-ray in another clinic [office], and come back.”

### Barriers to Oral Health Care Access in Canada

Participants reported facing many barriers to access to oral health care in Nova Scotia. Significant barriers included cost, long wait lists, knowledge gaps (lack of understanding of oral health care services and dental insurance system), and cultural differences.

#### Cost

The cost of oral health care in Nova Scotia was a significant barrier for all participants. They expressed having many competing financial priorities while trying to resettle in a new country. Solomiia mentioned,

The price [treatment cost] is the main barrier. I believe that accessing oral health care services is the basic aspect of our lives. The person should feel themselves safe. [Ukrainian newcomers] shouldn’t think about where to get money from in case of tooth loss. I believe that half of people cannot afford oral health care here.

Many considered dental care cost to be so high in Canada that they would travel outside Canada if they required extensive dental care such as prosthetics (crowns and implants). Yuliia stated, “Heaven forbids, I will not need a dental crown or teeth pulling out, heaven forbids. If it happens, I will go to Ukraine buying a ticket for CAD $800. It will be cheaper than here.”

It is important to note that having dental insurance was viewed positively, as insurance can help reduce some of the financial burden of seeking oral health care, making it more affordable for newcomers. However, individuals may have to delay dental treatment while waiting for dental benefits to become available. As Ivan explained,

I explained to [OHCPs] that I could come in two months only and asked if it would be an issue. They said that I could wait, but it would be better to have treatment as soon as possible. When I received dental benefits, I went and proceeded with treatment of my nonurgent problems.

For many people, dental benefits could be limited or be exhausted before all required dental treatment has been completed. Sofiia expressed, “But [dental benefits] ended. Now it is very expensive for me.”

#### Scheduling and Wait Times

Perception of timely access to oral health care varied from participant to participant, depending on oral health care needs and family status. While some participants felt that access to oral health care in Nova Scotia was relatively easy, the majority expressed frustrations with wait times for scheduling appointments. On the ease of scheduling a dental appointment, Kateryna commented, “Getting the appointment was not so hard. I scheduled it the week before. I didn’t wait too long.” Yuliia’s experience differed though:

Everything is not fast here. Once, I tried to schedule an appointment. People were on vacation, then there were some holidays. When I finally reached the clinic, the appointment was scheduled only in 2 months. I did not have any problems. I wanted to have a prophylaxis visit and order dental cleaning.

Six participants expressed frustration with the inflexibility of the OHCP’s schedule. This was a further inconvenience as it required taking time off work for appointments. Sofiia stated,

Now I need to adapt to the dentist’s schedule. In Ukraine I could choose the days by myself. Here I should make the appointment a month before it. Some days are not available for me. Also, I needed to ask for leave from work to have a dental visit. I adapt for the dentists; the dentists do not adapt for me.

#### Lack of Understanding of Oral Health Care System

Many expressed concerns about accessing specialized oral health care in Nova Scotia due to a lack of understanding of the referral process, different types of OHCPs and their scope of practice, and lack of specialist’s services in rural/remote locations. They did not understand why they could not directly contact and book an appointment with the specialist that they required. Oksana stated,

The dentist in Windsor [town in Nova Scotia] told us that the root canal treatment can be done in Halifax [capital city of Nova Scotia]. We expected that after the root canal treatment, the dental filling should be placed for my husband in this place [same dental office] as well. We wanted to book an appointment for it. But the dentist there told us that they are specialized only in root canal, and we should find another doctor for having a dental filling. It was . . . [smiles with surprise]. It is acceptable if you live in Halifax. But if you live in the Valley [region of Nova Scotia], you are attached to Halifax for specialized dental procedures.

Yuliia added, “The cleaning was not done by a doctor. It was maybe the assistant . . . it could be the dental hygienist. Maybe, she was a doctor’s assistant or a nurse as I understood.”

Those who lived in rural areas of Nova Scotia faced significant barriers to accessing urgent oral health care services for their children for dental pain. In most cases, they had to travel to the capital city, far from their residence, adding further challenges. Oksana explained,

We have faced the question of dental treatment for children, for example such one, which is needed for my son. The only decision is to go to Halifax. It is okay if you have a car. But it is not okay if the child experiences acute pain. In the Valley, if the children need dental emergency case treatment, the only decision is to give them painkillers. There are dentists there. But they do not treat such difficult cases, in which there is tooth pain.

#### Lack of Knowledge of Dental Insurance System

While having dental benefits was reported as important, many did not understand how dental benefits work. Dental benefits were not common in Ukraine, and having never utilized such a system before, participants reported confusion and frustration in navigating the process. Oksana mentioned,

People with dental coverage plan do not understand how it works. It is important. Nobody explains it. We go to the dental clinic [office] on our own. At the reception, we accidentally learned that dental coverage allocates to the amount of money for treatment, and it is for the year for example. Generally, we do not have any information about how dental insurance works. Canadians do not provide us with this information. Not because of they do not want to share information, they have been living with the insurances all their lives. They are obvious things for Canadians. They do not understand why it should be explained. It is very important to mention it because we do not have insurance system in Ukraine. We are not aware of its mechanism.

Some participants described being unsure about the extent of their dental benefits and their coverage or how to access it. This resulted in unexpected out-of-pocket expenses for dental treatments. Sofiia explained,

Once, after the dental visit I went to the reception. Usually, I did not pay or pay only a certain small amount. And the whole amount, approximately CAD $560, was announced for me. I asked why. They told me that my dental benefits were over. This should be told in the beginning of the visit. I had to pay with a credit card. I had a debt after it.

Additionally, there were misconceptions and misunderstandings about the employment-based dental benefits extending to family members. Sofiia added, “It was explained that only one spouse can have dental benefits at my work. My husband’s benefits are better. I can’t use both.”

#### Lack of Information for Oral Health Care Resources

Many faced difficulties accessing oral health care due to a lack of information about the available oral health care services and where and how to seek emergency dental treatment. Oksana mentioned, “[Ukrainian newcomers] hear from other Ukrainians about the expensive oral health care service in Canada and difficulties with accessing it. If Ukrainians have any emergency dental situation, they do not know what to do. They do not know where they can remove the tooth in case of emergency.”

#### Language-Culture

Language and cultural barriers affected many participants with limited English language proficiency while seeking oral health care in Nova Scotia. This language barrier made communication with OHCPs difficult and reduced their ability to understand processes such as treatment procedures and effective utilization of dental insurance benefits. Sofiia explained, “But first of all, it is difficult for me, because I do not know English. If I knew it, I would learn about benefit usage easily. I had difficulties with communication. . . . My level of English is not enough.”

### Facilitators for Accessing Oral Health Care Services in Ukraine

Most participants acknowledged ease of scheduling oral health care appointments and cost (affordability) as notable facilitators for accessing oral health care services in Ukraine. The changes in oral health care culture after Soviet Union times and dentists proficient in a number of specialties also contributed to oral health care access.

#### Ease of Scheduling Appointments

Most reported that oral health care services in Ukraine and scheduling appointments to see a dentist were convenient. The wait times were within a few days to a week, and in emergency situations, they could often have a visit and treatment on the same day. Ihor stated, “The access is easy. When you have an emergency case, you may even have a visit to my dentist’s clinic [office] the same day. For regular visits, I usually needed to wait for 1–1.5 weeks.”

#### Affordability

Most participants described oral health care as affordable, pointing out that the price for consultation and treatment was reasonable. Solomiia expressed,

I did not receive a large income. But I could afford payment for dental services—dental filling placement with good quality materials, dental surgical operations, etc. I have never been shocked by the price. My husband and I are average people within income level. We could even have covered the cost of prosthetics with our salaries if I had time [before leaving Ukraine due to war].

#### Oral Health Care Culture Shift after Soviet Union Times

The transformation of oral health care culture with the introduction of privately owned dental offices after Soviet Union times had a notable impact on the utilization of oral health care services. The increased use of modern materials, equipment, and anesthesia improved the quality of care. Yuliia reported, “When the Soviet Union was finally destroyed, step by step anesthetics appeared in dental practice.” Andriy also expressed, “You know before the development of private dental practice, new materials, new equipment and new medicines, everything was awful. Black lead fillings, unclear modeling was used.”

#### Full-Service Dental Offices

Most reported high levels of satisfaction with visiting dentists in Ukraine, who were proficient in different specializations. Participants expressed gratitude for their dentists’ skills, pointing out their ability to perform a range of procedures effectively and efficiently. Ihor reported, “He [dentist] is well skilled in a variety of dental fields. I have never had any complaints about him. My dentist was specialized in different field—therapeutic, surgical dentistry.” Oksana also commented, “My dentist gave me a lot of advice. He is very multifunctional—the dentist performed dental filling placement, prosthetics, dental implantation well for me.”

### Facilitators for Access to Oral Health Care Services in Canada

Employment-based dental insurance, proximity to dental offices, social networks, and language interpreters were facilitators for accessing oral health care services in Nova Scotia.

#### Dental Insurance Benefits

Dental insurance benefits helped ease the cost of oral health care to make it affordable. Maksym described, “Prices here do not hit the pocket for newcomers, who have a good job with dental benefits. Benefits covered 80%. I was satisfied with it. It was affordable for me.”

#### Social Networks, Location of Dental Offices, and Interpreters

Some facilitators for locating OCHPs included recommendations from family, social media, and workplaces. Kateryna stated, “I took advice from my son. He has been visiting dentists here for the long time. He knows the contacts of the dentists, whom I visited here. They are located in the same building as ISANS, at Mumford.”

Another facilitator was the proximity and number of dental clinics available. Maksym expressed, “We used geolocation to find the nearest clinic. We went there and stayed there [proceeded with treatment].”

Miscommunication resulting from a lack of English language proficiency was significantly reduced with the presence of language interpreters in dental offices. Sofiia, who was very confused about her sons’ dental treatment due to the language barrier, mentioned, “I was lucky to have an interpreter in the second clinic. It was not the same when I was in the dental clinic with my sons.”

### Suggestions for Improving Oral Health Care Access for Newcomers

When asked about their perceptions of what changes would help access to oral health care services in Canada for newcomers, most made several suggestions. These included financial assistance in the form of discounts or free initial oral examinations, information on the Canadian oral health care system, assistance from intermediates (ISANS, YMCA, Dalhousie University), and recruitment of Ukrainian OHCPs.

#### Discounts

Most participants were concerned with the high cost of oral health care services in Nova Scotia and suggested strategies for making these services more affordable. Maksym stated,

Maybe some discounts for people without insurance should be done for the first time. It is very difficult to pay CAD $500 per tooth. It is very difficult to with minimum salary CAD $16–17 per hour. Maybe, the benefit example can be used and newcomers from Ukraine can pay 20% of treatment during the first year. During the year they may find a good job with normal insurance.

#### Cost-free or Reduced-Fee Consultation

All participants consistently expressed a strong need for free oral health care consultations. They pointed out the importance of these consultations as a starting point for improving oral health care access for Ukrainian newcomers as they restart their lives after fleeing their homes and seeking refuge in Nova Scotia. Many participants believed that a full dental examination would allow individuals to learn about their oral health status, understand their treatment needs, and make informed decisions about their oral health care. Solomiia mentioned,

Ideally, the consultation could be free. The doctor should examine the oral cavity and tell the current oral health status. If for example 5, 6, 14 teeth have cavities, the dentist should inform about the need in dental filling and tell the price. If the doctor suggests x-ray for further diagnosis, x-ray should be paid of course. But the consultation should be free. So, the person could know their oral health issue, come home and think if it is affordable and plan the treatment.

#### Informing Ukrainians About Oral Health Care System in Canada

Most did not understand how to access oral health care works in Canada. They expressed a clear need to be informed on dental insurance, emergency dental care, and locations of dental offices and suggested including information sessions or webinars and creating guides specifically tailored for Ukrainians to navigate oral health care services. Oksana stated,

If it is possible, provide Ukrainians with information about dental coverage mechanisms, contacts for getting emergency dental care for adults and children. Also, about the information session—it can be the webinar for Ukrainians. The relevant questions should be gathered. It will help to prepare the presentation. After the presentation a Q and A time should be available. It is necessary to include the information about the insurance in the webinar.

#### Receiving Oral Health Care with Assistance of Intermediates

The participants recommended involving organizations such as resettlement organizations and universities with dental programs to allocate funds for covering specific dental procedures, providing information about oral health care system in Nova Scotia, and simplifying appointment processes. Kateryna reported,

Dental cleaning could be covered by resettlement organizations. It can be applied to adults in this case. Such organizations may not cover completely, but maybe 70% could be allocated from their funds. It will be the good practice. It makes difference if you pay CAD $30 or CAD $100.

Additionally, they suggested partnerships with universities that offer degrees in dentistry where dental students could offer cost-effective oral health care services. Kateryna added, “Getting dental care at the universities could be the perfect option. Students may provide dental care for Ukrainian newcomers. I believe it may be cheaper. Moreover, the students will get more practice, so it will be useful for them.”

#### Recruitment of Ukrainian OHCPs

Participants noted the importance of increasing the number of Ukrainian dentists in Nova Scotia. Ukrainian newcomers would benefit from access to OHCPs who have the same cultural background and can speak their language. Solomiia mentioned, “[Having Ukrainian dentist] will ease ‘dental adaption’ for patients. I believe that every Ukrainian newcomer dreams to visit the compatriot [same nationality] as the dentist.”

## Discussion

This descriptive qualitative study explored Ukrainian newcomers’ experience with accessing oral health care services in Ukraine and Nova Scotia. To our knowledge, this is the first study conducted with Ukrainian newcomers who arrived in Canada after fleeing the war. Most of the barriers to accessing oral health care services in Ukraine, such as personal safety and power outages, were due to the war. In contrast, affordability, lack of information on how oral health care services work, and wait times were significant barriers to accessing oral health care services in Nova Scotia. Providing information on the oral health care system, dental insurance benefits, discounts or payment plans, and language interpreters could facilitate access to oral health care services for newcomers. A concept map illustrates the connection of the barriers and facilitators to access of oral health care (Figure).

**Figure.**
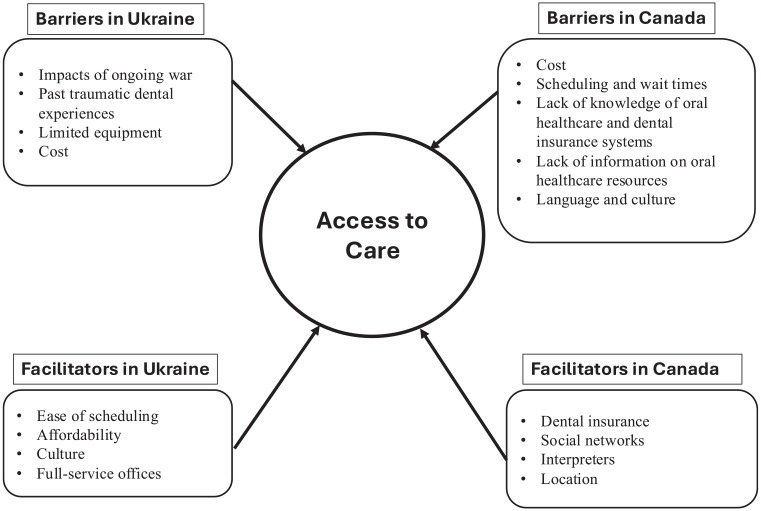
Concept map: barriers and facilitators to care.

To provide the proper context when discussing the barriers and facilitators of access to oral health care in Ukraine, it is necessary to explain the current Ukrainian dental care system. Prior to the Ukrainian Declaration of Independence in 1991, dental care was provided through the public sector. Since this time, health care and oral health care have gone through a transition to a mix of public (state) and private practices. There has been a decrease in the number of, as well as insufficient funding for, state-run clinics, which has affected access to oral health care for the general population (Chopchyk et al. 2021). This has led to a high level of oral disease in the population when compared with other European countries, with many Ukrainians being unable to access state clinics and afford oral health care in private clinics (Chopchyk et al. 2021). While the majority of participants in our study found oral health care affordable in Ukraine, rising prices and income-related challenges were highlighted by some. Participants in this study may represent a financially stable segment of the general population’s income levels, which could influence perceptions of affordability.

Although access to oral health care may have been problematic to some prior to war, the war disrupted oral health care utilization, leading to missed appointments, treatment delays, and challenges in completing essential procedures such as placement of prosthetics. Additionally, safety concerns and power outages compounded the obstacles faced by participants. These findings are in alignment with reports that the ongoing war has affected access to all health care services in Ukraine through security concerns, restricted mobility, broken supply chains, and lack of running water or electricity (Dzhus and Golovach 2023).

Willingness to access oral health care services in Ukraine for participants aged ≥38 y was influenced by previous traumatic oral health care experiences from the Soviet Union era, where local anesthesia was often unavailable or not utilized for painful procedures, contributing to a persistent fear of dental visits and avoidance of oral health care. It has been well established in the literature that previous negative experiences when accessing oral health care can lead individuals to avoid seeking care except in emergency situations (Rajeev et al. 2020). This influences the need for easy access to oral health care in countries of resettlement as challenges may further deter desire to seek care, thus contributing to poor oral health outcomes and reduced quality of life.

In contrast to the literature that speaks of lack of access to oral health care professionals in Ukraine (Chopchyk et al. 2021), participants in this study reported facilitators for oral health care access in Ukraine to include the ease of scheduling appointments and affordability. Most participants commented on convenient and timely access for regular and emergency visits. Many stated that oral health care was reasonably priced and accessible, and they expressed satisfaction with the cost of consultations and treatments. While this contrasts the available literature, it is important to take into consideration the demographics of the participants in this study, whose experiences may have been the result of living in one of the major centers in Ukraine where access to state and private clinics would have been better than in smaller or more rural areas.

Participants who were able to recall oral health care experiences during the Soviet Union era commented on significant improvements in oral health care post–Soviet Union, through the introduction of privately owned dental offices, modern materials, equipment, and anesthesia. Currently in Ukraine, there are 8 recognized dental specialties. The specialization period tends to be from 3 to 9 mo depending on the field (Ministry of Health of Ukraine Order 2003). It may be beneficial for dentists to obtain specializations to be able to provide a range of dental treatment procedures. What participants perceived as “full-service dental offices” contributed to high levels of satisfaction, with participants appreciating dentists who were proficient in various specializations, allowing for a range of effective and efficient procedures in 1 office as opposed to visiting specialists.

The oral health care system in Canada is organized differently than in Ukraine. Currently in Canada, there are 10 recognized dental specialties with varying lengths of time required that are significantly more than those in Ukraine (Canadian Dental Association 2024). General dentists may perform some of the same procedures as specialists, such as root canals, but many general dentists prefer to refer patients to specialists rather than completing the procedures themselves.

While the participants’ overall perspective of access to oral health care in Ukraine was a positive one, the view of access to oral health care in Nova Scotia was predominantly negative with Ukrainian newcomers reporting facing significant challenges. Similar to a recent study by Nurelhuda et al. (2021), which examined oral health care access for humanitarian migrants to Canada, cost emerged as a major barrier, with financial constraints and limited dental insurance coverage affecting the ability to afford necessary treatments. Oral health care in Canada is predominantly based in private dental settings, with 56% of the cost covered by private insurance and 38% by out-of-pocket expenses, resulting in 1 in 6 Canadians reporting cost as the major deterrent in seeking out or proceeding with required oral health care (Yujiro and Roger 2022). Newcomers to Canada face additional challenges, such as labor market challenges leading to economic vulnerabilities including poverty, low income, underemployment, and unemployment (Beiser et al. 2002; Sakamoto et al. 2010). For participants in this study who had employment-based oral health care insurance, the satisfaction with and affordability of oral health care in Canada were notably improved as it provided substantial financial assistance for oral health care costs. While the Canadian Interim Federal Health Program provides basic dental coverage for government-assisted refugees, displaced Ukrainians are not eligible for this program. Access to this basic dental coverage may have eased out some of the challenges faced by Ukrainians newcomers.

Scheduling appointments proved challenging, with varying wait times and the inflexibility of OHCPs’ schedules causing frustration, particularly for those needing to align visits with work commitments. It has been noted in the literature that new immigrants and refugees to Canada have difficulty accessing health care for themselves or their children, as most appointments require taking time away from employment, which in many cases may not be possible (Salami et al. 2020; Dahal et al. 2024). Taking time away from school or work leads to productivity loss and can significantly affect their resettlement in a new country.

Knowledge gaps were evident, with participants struggling to understand the referral process for oral health specialists and the utilization of dental insurance benefits. While participants appreciated the dental insurance benefits, in some cases the lack of understanding the details of the insurance led to unexpected out-of-pocket expenses and added financial debt that required using money reserved for basic living expenses. Lack of understanding of the health and oral health care systems by newcomers to Canada has been discussed as a significant hurdle in access to care (Salami et al. 2020; Nurelhuda et al. 2021; Yujiro and Roger 2022).

Language is integral to an individual’s identity and is reflective of culture (Jiang 2000). It has been well established in the literature that health outcomes are improved when individuals receive care in their own language (Flores 2005; Itaya et al. 2009). Language and cultural barriers were discussed as being multifaceted, impeding effective communication with OHCPs and contributing to a lack of understanding of oral health care procedures, referrals, and dental insurance. This finding aligns with studies that discuss how limited English language proficiency makes it difficult for newcomers to obtain information regarding recommended oral home care practices and details of dental insurance and navigate access to oral health services (Dong et al. 2011; MacEntee et al. 2012; Jang et al. 2014; Luo and Wu 2016; Doucette et al. 2023; Ponomarenko and Kaifie 2023). Literature speaks to the need for oral health professionals to increase their cultural competence in an effort to minimize the barriers to oral health care for newcomers (Patrick et al. 2006; Batra et al. 2019; Crespo 2019; Keboa et al. 2019).

For those participants who were fortunate to have language interpreters available, the presence of interpreters significantly reduced miscommunication arising from language barriers, ensuring effective communication and enhancing access for individuals with limited English language proficiency. While the use of language interpreters in the health care system is mandated in the United States (Joint Commission on Accreditation of Healthcare Organizations 2012), across Canada interpretation services in health care settings are delivered by provincial agencies, regional health authorities, and individual health care organizations and are mandated by law only for American Sign Language and French. There is no equivalent law for health care delivery in other languages (Sultana et al. 2018). The placement of oral care outside of universal health care plans in Canada means that the responsibility of arranging and financially compensating interpreters lies with individual OCHPs or the patient, resulting in underutilization of interpretation services (Amin et al. 2014).

Social networks, including familial recommendations, played a main role in guiding individuals to OCHPs. This finding is in keeping with previous studies where immigrants and ethnic minorities who had a large social network and frequent discussions were more knowledgeable about oral health care services and oral health knowledge, which resulted in increased oral health care utilization (Dahlan et al. 2019). The accessibility of dental clinics, facilitated by geolocation and geographic convenience, served as a critical facilitator in enhancing oral health care access. Workplace recommendations underscored the influential role of professional networks in assisting newcomers in navigating the Canadian oral health care system.

Ukrainian newcomers to Nova Scotia provided suggestions for improving oral health care access. Concerns about the high cost of oral health care services led to recommendations for financial assistance, including discounts and free initial oral examinations. The need for cost-free or reduced-fee consultations was emphasized, with participants highlighting the importance of understanding oral health status and treatment needs. To address the lack of awareness about the Canadian oral health care system among Ukrainian newcomers, participants proposed informative sessions, webinars, and tailored guides covering topics such as dental insurance and emergency care. Involving intermediaries such as ISANS, YMCA, and universities, particularly Dalhousie University Dental Clinic, was suggested. It was also suggested that these intermediaries could allocate funds for oral health care for newcomers, help with simplifying appointment processes, and in the case of university dental clinics, offer cost-effective services. Finally, participants pointed out the importance of recruiting Ukrainian OCHPs to Nova Scotia to enhance cultural understanding and language accessibility.

## Limitations

It is important to shed light on the following limitations while interpreting the study findings. The findings reflect the perceptions of specific Ukrainian newcomers who participated in the study. To overcome this, of the 31 who expressed interest in participating, we purposefully selected 10 participants with varied backgrounds. However, those who expressed interest in participating in the study could be different from those who did not want to participate. It is possible that most participants were frustrated with seeking access to oral health care services as newcomers and wanted to provide input to improve the experience for other newcomers. It was not our intent to generalize the findings to all newcomers in Canada but a specific group of newcomers who arrived in Nova Scotia on humanitarian grounds without the government-provided oral health care benefits available to other classes of refugees. Thus, our goal was to describe the experiences of Ukrainian newcomers in accessing oral health care services in Nova Scotia.

## Conclusion

Ukrainian newcomers face significant barriers to access of oral health care services in Nova Scotia. The most reported barriers were cost, lack of knowledge of the oral health care system, language, and culture. While many Canadians may face similar issues, Ukrainian newcomers have additional unique challenges surrounding resettlement in a new country that compound these barriers. As quality of life is directly related to oral health and oral health care access is linked to oral health outcomes, it is important that policies and practices be put in place that are aimed at addressing these barriers.

## Author Contributions

H. Doucette, contributed to conception, design, data acquisition, analysis, and interpretation, drafted and critically revised the manuscript; Y. Tylchak, contributed to conception, design, data acquisition, analysis, and interpretation, drafted aspects of the manuscript, reviewed the drafted manuscript and provided revisions; S. Saad, contributed to data analysis, reviewed the drafted manuscript and provided revisions; V. D’Souza, contributed to conception, design, data acquisition, analysis, and interpretation, reviewed the drafted manuscript and provided revisions. All authors gave final approval and agree to be accountable for all aspects of the work.
